# Interventions employed to address vaccine hesitancy among Black populations outside of African and Caribbean countries: a scoping review

**DOI:** 10.1186/s12889-024-20641-3

**Published:** 2024-11-13

**Authors:** Precious Majekodunmi, Mia Tulli-Shah, Janet Kemei, Ibukun Kayode, Aloysius Nwabugo Maduforo, Bukola Salami

**Affiliations:** 1 Faculty of Nursing, University of Alberta, Edmonton, AB Canada; 2 Black and Racial Health Equity Research Program, Department of Community Health Sciences, Cumming School of Medicine, University of Calgary, Calgary, AB T2N 4Z6 Canada; 3 Faculty of Nursing, MacEwan University, Edmonton, AB Canada; 4 Interdisciplinary Studies Graduate Program (ISGP), University of British Columbia, Vancouver, BC Canada; 5 Centre for Migration Studies, University of British Columbia, Vancouver, BC Canada; 6 Werklund School of Education, University of Calgary, Calgary, AB Canada

**Keywords:** COVID-19, SARS-CoV-2, Vaccine hesitancy, Vaccine confidence, Black populations, People of african descent, Pandemic

## Abstract

**Background:**

Black people are disproportionately affected by structural and social determinants of health, resulting in greater risks of exposure to and deaths from COVID-19. Structural and social determinants of health feed vaccine hesitancy and worsen health disparities.

**Objective:**

This scoping review explored interventions that have been employed to address vaccine hesitancy among Black population outside of African and Caribbean countries. This review provides several strategies for addressing this deep-rooted public health problem.

**Methods:**

The scoping review followed the five-step framework outlined by Arksey and O’Malley. It complies with reporting guidelines from the Preferred Reporting Items for Systematic reviews and Meta-Analyses extension for Scoping Reviews (PRISMA-ScR). Research studies that examined interventions utilized to promote vaccine confidence within Black populations living outside of African and Caribbean countries were reviewed.

**Findings:**

A total of 20 articles met the inclusion criteria for this study: 17 were quantitative studies and three were mixed-method studies. This scoping review highlighted six themes: educational advancement, messaging, multi-component approaches, outreach efforts, enhancing healthcare access, and healthcare provider leadership.

**Conclusion:**

The review identified effective interventions for addressing vaccine hesitancy among Black populations outside Africa and the Caribbean, emphasizing education, multidimensional approaches, and healthcare provider recommendations. It calls for more qualitative research and interventions in countries like Canada and the UK to enhance vaccine confidence and reduce mistrust.

**Supplementary Information:**

The online version contains supplementary material available at 10.1186/s12889-024-20641-3.

## Introduction

“Vaccine hesitancy” now falls under the motivation domain and is defined as a state in which individuals feel conflicted or opposed to getting vaccinated, encompassing their intentions and willingness [1]. This updated definition replaces the 2014 description by SAGE, which characterized vaccine hesitancy as a delay in accepting or refusal of vaccination despite its availability [2]. The revised definition emphasizes hesitancy as a motivational state, distinct from the resulting behaviour. This approach allows for a clearer understanding and measurement of behaviors and their diverse influences, treated separately from underlying motivation. Vaccine hesitancy emerged as a significant global health challenge, particularly in the wake of the COVID-19 pandemic. This phenomenon is complex and context-specific, varying across time, place, and vaccines [3]. Among Black populations outside of African and Caribbean countries, vaccine hesitancy is influenced by a myriad of factors including historical injustices, mistrust in healthcare systems, misinformation, and socio-economic barriers [4–6]. Data from the United States reveal that Black populations have a higher prevalence of COVID-19 infection and death from the infection, partly due to greater representation in service occupations and higher likelihood of living in densely populated inner cities [7, 8]. Further data suggest this may be attributed to pre-pandemic vulnerability of developing hypertension, asthma, and chronic diseases among Black Americans, which further heightens their risk of comorbidities and results in worse COVID-19 outcomes [8, 9]. A 2021 census from the United Kingdom indicates Black people had the highest rate of death from the COVID-19 virus, with this rate being 3.7 times greater than for their white counterparts [10]. In Canada, a lower proportion of Black Canadians (56.6%), compared to white Canadians (77.6%) reported being somewhat willing to receive a COVID-19 vaccine [11].

Running parallel to a disproportionate vulnerability to COVID-19 infection is rates of uptake of existing vaccines such as human papillomavirus (HPV), influenza, and COVID-19 that remain low among Black populations living outside of Africa and Caribbean countries. Disparities in immunization rates extend beyond COVID-19, with Black adults being less likely to receive vaccines for influenza, HPV, or Tdap [7, 12]. For instance, during the 2019–2020 flu season, only 35% of Black adults in the United States received an influenza vaccination compared to 55% of their white counterparts [13]. Addressing vaccine hesitancy in these communities is crucial for improving vaccination uptake and ensuring equitable health outcomes [12].

The low uptake of existing vaccines among Black populations living outside Africa and the Caribbean indicates the need for interventions to promote vaccine confidence and address vaccine hesitancy among this population. Numerous determinants such as complacency (perception of a low risk of an infection or low efficacy), confidence (lack of trust in vaccines, the healthcare provider, vaccine manufacturer, and public health system), and convenience (barriers to vaccination) contribute to vaccine hesitancy globally [13]. Black communities in Canada and the United States have begun utilizing an Afrocentric health promotion approach (Afrocentric health promotion refers to health education and intervention strategies that are specifically designed with the cultural, historical, and social contexts of people of African descent in mind. This approach aims to make health messages, resources, and interventions culturally relevant and resonant with Black communities, recognizing unique health beliefs, practices, and experiences within these populations) and mobilizing to deliver workshops, town halls, free vaccine clinics, and social media campaigns to address vaccine hesitancy among Black populations, especially in response to COVID-19 [14–16]. However, these efforts should be conducted utilizing research evidence and guidelines on strategies to effectively improve vaccine uptake among people of African and Caribbean descent [14, 17, 18].

Given the critical need to enhance vaccine acceptance, various interventions have been implemented to address the unique challenges faced by Black populations in diaspora settings. These interventions range from community engagement and education campaigns to policy changes and healthcare provider training [5, 19]. However, the effectiveness of these interventions varies, and a comprehensive understanding of what works, for whom, and under what circumstances is essential for designing effective strategies [20]. This scoping review aims to map the existing literature on interventions employed to address vaccine hesitancy among Black populations outside of African and Caribbean countries. By systematically reviewing and synthesizing the evidence, this study seeks to identify the types of interventions implemented, their outcomes, and the contextual factors that influence their success. The findings will provide valuable insights for policymakers, healthcare providers, and community organizations striving to improve vaccine uptake in Black communities and reduce health disparities [21–23].

## Methods

A scoping review was conducted to explore interventions designed to address vaccine hesitancy among Black populations outside of African and Caribbean countries. Arksey and O’Malley’s five-stage methodology [24] was selected for this review due to its comprehensive framework for mapping existing literature and identifying research gaps, making it particularly suitable for exploring diverse interventions and outcomes. This approach allows for a systematic examination of studies focused on strategies to promote vaccine confidence within Black populations, providing a structured yet flexible method to address the complex factors influencing vaccine hesitancy.

### Stage 1: identification of research question

The following questions guided this review: (1) What is the extent and nature of the literature on the interventions to address vaccine hesitancy among Black populations worldwide with the exclusion of studies based in African and Caribbean countries? (2) What are the gaps in evidence on the vaccine hesitancy interventions targeting Black populations living outside of African and Caribbean countries?

### Stage 2: identification of relevant studies

A health science librarian assisted in refining our search strategy and in searching the following databases: Medline, Embase, PubMed, Cumulative Index for Nursing and Allied Health Literature (CINAHL), ProQuest Sociological Abstracts, and Scopus via Elsevier. The search strategy was performed using the following keywords: (1) vaccine hesitancy and vaccine confidence; (2) interventions or strategies or approach or methods or campaign; (3) vaccination or immunization; (4) vaccine acceptance; (5) Black populations or people of African descent, or African immigrants; (6) African Americans or Black Americans; (7) North America or United States or Canada; (8) United Kingdom or England or London; (9) Australia; and (10) Asia. No publication date, study type, or language restrictions were applied to the literature search.

We used Covidence, a web-based software platform designed to streamline the process of conducting systematic reviews. It offers features such as citation screening, full-text review, risk of bias assessment, and data extraction, making it easier for researchers to efficiently manage large volumes of literature. Covidence supports collaborative review by allowing multiple reviewers to work simultaneously [25]. A total of 3835 articles were retrieved from the above databases, with 2786articles remaining after duplicates were removed. To remove duplicate records and facilitate screening, records from each database search were exported in complete batches and added to Covidence. We also used Google searches to further shed light on interventions that are tackling vaccine hesitancy among Black populations. Four articles from the Google search that fit the inclusion criteria were included in the article screening. We also conducted a gray literature search, which included searches of the websites of government agencies as well as organizations that focus on Black health, population health, distrust of vaccines, and vaccine reluctance. Additional studies were identified through a scan of the reference lists of articles selected as meeting the study inclusion criteria. The search was completed May 20, 2023 Table 1.

**Table 1:** Search strategy

Search Strategy Component	Details
**Databases Searched**	Medline, EMBASE, PsycINFO, CINAHL, Cochrane Library, Scopus, Web of Science, ProQuest Dissertations and Theses, Sociological Abstracts
**Keywords and Boolean Operators**	
Core Concepts	1. Vaccine hesitancy and vaccine Confidence or trust
	2. Interventions, strategies, approach, methods, or campaign
	3. Vaccination or immunization
	4. Vaccine acceptance
	5. Black populations, people of African descent, or African immigrants
Population Keywords	African Americans or Black Americans
Geographical Focus	1. **Canada and Provinces/Territories**: Canad*, “British Columbia”, “Colombie Britannique”, Alberta*, Saskatchewan, Manitoba*, Ontario, Quebec, “Nouveau Brunswick”, “New Brunswick”, “Nova Scotia”, “Nouvelle Ecosse”, “Prince Edward Island”, Newfoundland, Labrador, Nunavut, NWT, “Northwest Territories”, Yukon, Nunavik, Inuvialuit
	2. **North America and U.S. Regions**: “North America*”, “United States”, USA, American*, followed by individual U.S. states (e.g., alabama or alaska or arizona or arkansas or california or colorado or connecticut or etc. (list all states)
	3. **Mexico and Mexican States**: Mexic* or aguascalientes or baja or campeche or chiapas or chihuahua or coahuila or etc. (list all mexican states)
	4. United Kingdom, England, London
	5. Australia
	6. Asia
Gray Literature	Conducted targeted Google searches using similar keywords and geographical terms to locate gray literature.
Date, Study Type, and Language Restrictions	No restrictions were applied for publication date, study type, or language, ensuring a comprehensive and inclusive literature review.

### Stage 3: selection of eligible articles

**Fig. 1:**
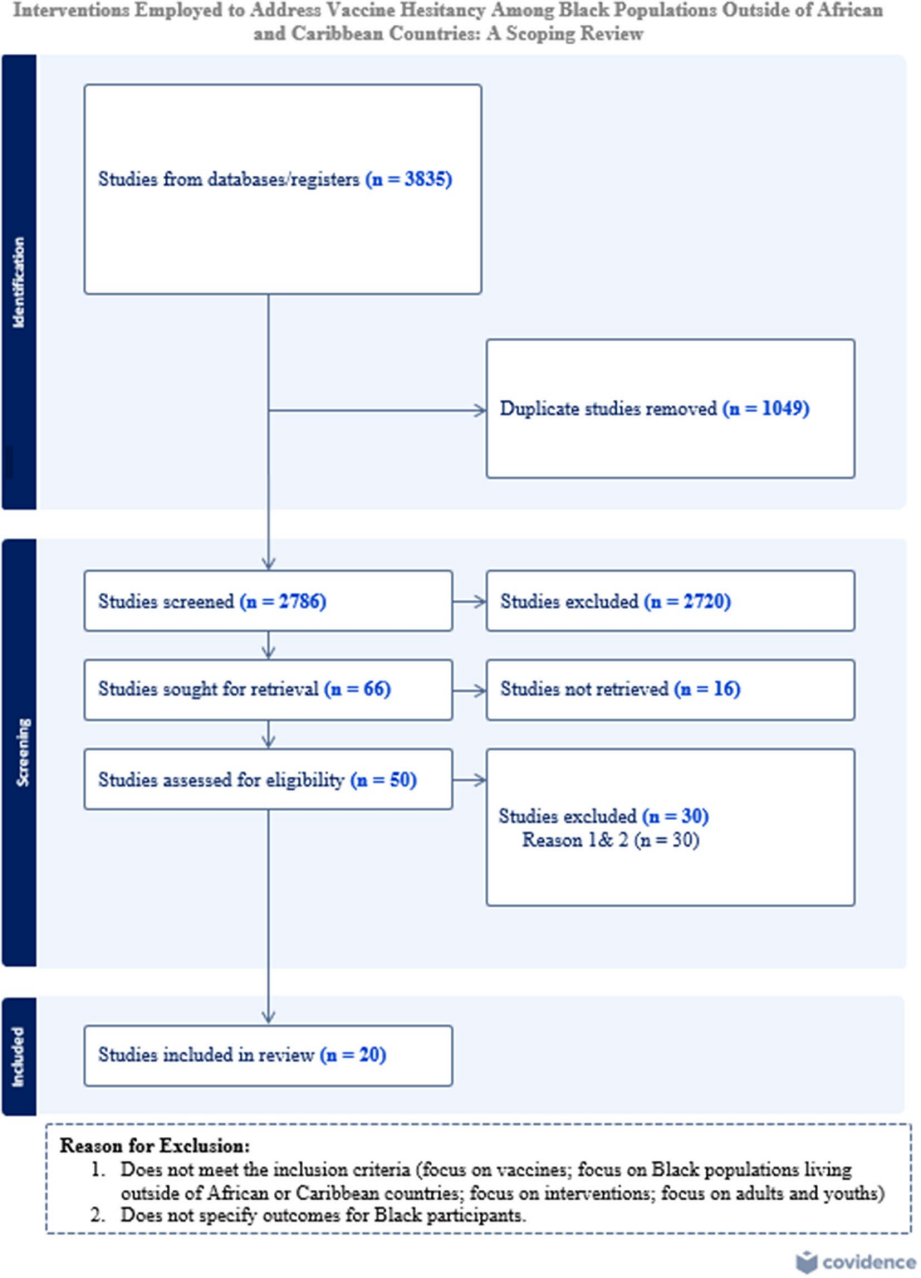
The scoping review procedure. Adapted from: [26]. The PRISMA 2020 statement

Two reviewers (PM and IK) independently screened the title and abstract of the 3835 articles yielded from the article search. They then conducted a full-text screening of relevant articles to identify those that met the inclusion criteria. A third member (JK) of the research team resolved any disagreements between the two reviewers at every article screening stage. Examples of disagreement were studies that did not focus solely on Black population. The inclusion criteria of this study were as follows: (1) focused on vaccine hesitancy, reluctance, confidence, or acceptability; (2) focused on interventions related to vaccines; (3) focused on Black people living outside of Africa or the Caribbean; and (4) focused on vaccines. During the screening, studies pertaining to Black populations worldwide with the exclusion of studies focused on African and Caribbean countries were included. Articles that did not specify the effect of the intervention on Black participants or did not discuss any vaccines were excluded. Interventions were excluded if they focused on improving vaccine uptake for the general population without including race-based data. Gray literature sources, including government reports, policy documents, and organizational publications, were carefully assessed using the established inclusion criteria, with particular attention to evaluating their quality and credibility given the variability in peer review and publication standards. We were unable to access the full version of 20 articles and hence excluded them as only an abstract or poster was available for review during the full-text screening. The article selection procedure is presented in Fig. 1.

### Stage 4: data extraction and charting

An Excel spreadsheet was used to document the data extracted from each article. The extracted data included the following characteristics: (1) author name and year of publication; (2) title of the study; (3) purpose of the study; (4) methodology; (5) sampling, recruitment, and selection process; (6) sample size; (7) type of intervention; (8) location; (9) results; and (10) implications. The accuracy of the data extraction was verified by a senior author (JK). This study did not include a quality appraisal of selected articles, as the main objective was to map out the extent of the existing literature.

### Stage 5: data analysis, summary and reporting of results

The study conducted a numerical summary and thematic analysis of included articles using Braun and Clarke’s six-step thematic analysis process [27]. The authors thoroughly read the included articles several times to familiarize themselves with the data. They then generated initial codes relevant to the research question. These codes were collated into potential themes, and all data relevant to each theme were gathered. The data for each theme were compared to the entire dataset to ensure consistency. In addition to thematic analysis, a numerical analysis of the selected studies was conducted through a descriptive count of the number of articles based on extracted data characteristics, such as methodology and location of the included studies.

## Results

Table 2 (Supplementary file) presents the details of each study included in the review, focusing on interventions aimed at addressing vaccine hesitancy among Black populations. Each entry outlines the study’s authors and publication year, title, methodology, sampling, intervention type, and key findings, along with the study’s implications. This comprehensive overview provides a clear view of the scope and outcomes of various interventions and allows for comparison of approaches and their effectiveness across different settings and population subgroups.

A total of 20 articles met the inclusion criteria of this study: 17 were quantitative studies [28–43] and three were mixed method studies [37, 44, 45]. The type and size of the selected sample varied according to the study design. The majority of the research studies selected were conducted in non-clinical settings [28, 30–33, 35–40, 43–46]. In studies that utilized mixed methods, various types of sampling techniques were used and sample sizes ranged from 115 to 13,942 participants. A total of six research studies (five quantitative and one mixed methods) recruited mainly women as participants, including college students, mothers, and pregnant women [33, 36, 39, 41, 42, 47].

Four studies were conducted using various media, such as videos, computers, ebooks, and iPads [30, 32, 34, 36] and two studies were conducted using telephone calls [29, 41]. Clinicians from various healthcare disciplines including medical students, nurses, physicians and nurse practitioners were involved in certain studies [32–34, 36, 42, 44, 47].

Six themes emerged from this review: educational advancement (*n* = 8), messaging (*n* = 5), multi-component approaches (*n* = 3), outreach (*n* = 3), enhancement of healthcare access (*n* = 3), and health care provider leadership (*n* = 5).

### Educational advancement

Eight studies addressed vaccine hesitancy among Black populations by implementing educational interventions aimed at improving vaccine literacy [28, 30, 32, 34, 36, 38, 39, 44, 45]. These interventions used various formats, including leadership seminars [28], educational videos [30, 32, 34], an educational forum [44], PowerPoint presentation [34], continuing education course for healthcare physicians [36], comic book [38] and lectures [39]. Videos were the most frequently used medium, with several studies demonstrating that participants’ vaccine knowledge improved after video-based education [30, 32, 34]. Several studies found participants’ knowledge of vaccines improved after the implementation of any type of educational intervention [28, 30, 32, 34, 36, 38, 39, 44, 45]. For instance, Alio et al. utilized leadership development seminars, which included a theological framework, to provide training for Black faith leaders and build their capacity to discuss HIV prevention methods and vaccine research [28]. The majority of the Black clergy members who participated significantly increased their understanding of HIV vaccine research and methods for promoting vaccination [28]. A few studies focused on enhancing the digital health literacy of Black participants by utilizing different forms of media to disseminate information about vaccines [30, 32, 34]. Digital mediums used included educational videos and a computer administered video presentation [30, 32]. Findings from Chapman and Diclemente revealed a higher acceptance of the HPV vaccine in younger age groups after the administration of an educational video [30, 32]. Additionally, Kriss et al. used a video that depicted physicians administering the Tdap vaccine to an adolescent African American female and delivered an electronic book consisting of information about the Tdap and influenza vaccines to participants [34]. Findings from Kriss et al. revealed that participants who viewed the electronic book had the highest rate of vaccination completion while 29% of women who viewed the video completed their Tdap vaccination [34]. The electronic book was a low-cost and effective method for increasing the vaccine education of African American populations.

Another intervention study that included a video utilized a different approach by adding culturally appropriate (A culturally appropriate intervention is a health or social program tailored to align with the cultural values, beliefs, practices, and needs of a specific population. This type of intervention respects and incorporates the Black community’s language, traditions, and social norms to make the program more relevant and accessible. Culturally appropriate interventions aim to enhance engagement, effectiveness, and acceptance by acknowledging and valuing the unique characteristics of the Black community) images and messaging that influenced the attitudes of East African mothers towards vaccinating their adolescent children [44]. The videos offered in this intervention depicted East African mothers interacting with culturally similar health care providers in the participants’ first language and included testimonials from mothers about the importance of HPV vaccination. After watching the videos, 75.5% of participants reported a willingness to get their child vaccinated and 86.4% of mothers were more likely to speak to their child’s doctor about getting the HPV vaccine [44]. The HPV knowledge of mothers also increased as 88.4% of participants correctly answered questions about the HPV vaccines.

While many studies focused on increasing knowledge and acceptance of vaccines among participants, one study focused solely on educating health care providers (HCPs) [36]. McFadden et al. utilized an online continuing education course with content that consisted of HPV vaccine information, demographic information, cultural humility, and information about the norms that may be shared among East African communities (including Somalia, Ethiopia, and Eritrea) to educate HCPs about strategies to effectively advise East African parents to get the HPV vaccine for their children [36]. The confidence of HCPs to make strong HPV vaccine recommendations to East African families increased from 68% pre-test to 98% post-test, and up to 94% of providers had the ability to address common parental HPV vaccine concerns. Conversely, two educational interventions including a comic book [38] and a one-hour lecture [39] targeted vaccination uptake among Black adolescents. The findings revealed that participants who viewed the comic book and completed the lecture could correctly answer questions about the vaccine post-intervention, indicating increased knowledge [38, 39].

### Messaging

This review revealed a myriad of messaging strategies that focused on vaccine knowledge, acknowledgement of historical mistreatment in medical research, utilization of same ethnicity health experts, benefits of getting the vaccine, negative consequences of failing to get vaccinated, and physician video messages regarding vaccination [31, 33, 43, 45, 48].

Dhanani et al. identified that, among Black participants, messaging that acknowledged the historical unethical treatment of Black Americans in medical research and emphasized current safeguards to prevent medical mistreatment were associated with significantly less vaccine hesitancy than the control group without any type of messaging [31]. The same effects were not observed for the other messages used in the study that provided general safety information about the vaccine or that emphasized the role of the vaccine in reducing racial inequities [31]. A study by Frew et al. discovered that participant intentions to vaccinate did not significantly increase after the disclosure of the positive outcomes of vaccination [33].

Several elements of messaging were found to increase vaccine confidence. For example, Alsan et al. found vaccine messages delivered by a Black physician slightly increased Black participants’ search of further information about the COVID-19 vaccine [48]. Similarly, Huang et al. determined the feasibility of including self-persuasion narratives within a vaccine message to improve vaccine confidence [43]. Participants viewed one of three messages: (1) a narrative that described how a character has changed their mind about the COVID-19 vaccine and received a vaccine dose; (2) a narrative without the character’s self-persuasion that featured a character who had a strong positive attitude from the beginning and did not struggle with the vaccine decision; and (3) bullet point information found on the CDC’s website that discussed the benefits, safety, and possible side effects of COVID-19 vaccines. Their findings showed the non-narrative message was most effective in affecting vaccine beliefs and equally effective as the self-persuasion narrative in enhancing vaccination intentions [43].

Additionally, Gadarian et al. tested the idea that messaging featuring endorsements of vaccination from two health experts of the same race may increase vaccine confidence; however, the study found no such correlation [45]. All of the research articles that fit the communication-centered theme included general vaccine information in addition with other types of messages and reported less apprehension about vaccines.

### Multi-component approaches

Three studies investigated the outcomes of implementing multiple approaches to mitigate vaccine hesitancy within African American communities. The multi-dimensional theme refers to interventions that consisted of more than one method to improve vaccine confidence. For instance, the relevant articles incorporated simultaneous activities such as outreach programs, implementation of educational materials, and vaccine distribution [29, 37, 41]. After the implementation of multiple strategies (such as virtual town halls (listening sessions), the formation of a clinician team to provide vaccine outreach efforts, an anonymous confidential hotline, healthcare workers’ staff hurdle, written educational materials, and vaccine stations), Black healthcare workers involved in the 2019 Chan et al. study conducted in the United Kingdom at University College London accomplished the highest vaccination completion rate despite Black Americans possessing low rates of vaccination [29]. Conversely, a mixed-method study in Los Angeles County, conducted between October 23, 2009, and December 8, 2009, comprising free vaccine clinics, vaccine message advertisements, and targeted outreach to the African American community, showed limited impact as vaccination rates remained low post-intervention. This study focused on the H1N1 vaccination response, distributing vaccines to nearly 200,000 people through public mass vaccination clinics to address disparities across racial and ethnic groups. Findings highlighted significant underrepresentation of African Americans in vaccination uptake, despite concerted efforts to overcome distrust and misinformation within the community [37]. Black participants were more likely to initiate an HPV vaccine after the completion of a multicomponent intervention consisting of an educational brochure and recall for vaccine doses [41]. The study participants were initially mailed an educational brochure encompassing perceived risks, vaccine efficacy, and safety measures for the HPV vaccine [41]. Subsequently, nurses contacted parents who declined the vaccine at their clinic visit 2 weeks later to remind them that Parkland hospital providers strongly recommend the HPV vaccine and offered to schedule an immunization appointment within the second component of the intervention [41]. Parents that were 4 weeks overdue for the second or third HPV vaccine dose also received a call from a nurse 4 weeks later, at which time the importance of receiving all three doses was emphasized and the participants were offered the chance to schedule another vaccine appointment. The recall intervention was found to encourage Black participants to receive their third vaccine dose as a result of a reminder and recommendation from a HCP. Black populations had a low rate of receiving the first vaccine dose; however, recalls to parents who were overdue for their 2nd and 3rd doses effectively elevated the dose coverage for both Black and Hispanic individuals [41].

### Outreach

Three studies were designed to extend information about vaccines to Black populations through the use of multiple types of media, vaccine sites, and focus groups [29, 35, 36]. In one case, a media campaign aiming to reach mainly African American populations was conducted by the California Department of Health Services (CDHS) in multiple counties [35]. The campaign entailed vaccine messages such as the availability of free shots, prenatal responsibility to get vaccinated, and toddler immunization schedules, in addition to the inclusion of images of Black peoples and provision of vaccine information sheets to HCPs for distribution during clinical visits [35]. Other methods of dissemination in the campaign included billboard ads, television ads, newspaper, flyers, pamphlets, and radio announcements. The outreach campaign effectively reached more than half of the study participants as 63, 51, and 43% of participants recalled seeing/hearing television ads, billboards, and radio ads and over 88% of Black participants remembered seeing at least one form of media-based immunization advertisement.

In addition, improved confidence in SARS-CoV-2 vaccines was attributed to the formation of a team of clinicians who conducted vaccine education and outreach activities within a hospital [29]. Chan et al. found clinical educators who shared similar cultural backgrounds as their fellow co-workers were perceived as relatable and increased assurance among employees because the educators addressed specific clinical questions about the vaccine [29].

Finally, a third study utilized focus groups to develop well-informed interventions that would alleviate the vaccine concerns of Black Americans [36]. McFadden et al. conducted focus groups in three languages (Somalian, Amharic, and Tigrinya) to collect information from East African mothers and executed another focus group consisting of providers that regularly serve East African community members [36]. The providers reported on (1) key strategies that providers currently use to communicate with patient families about the HPV vaccine; (2) perceived barriers to HPV vaccine uptake for both East African families; and (3) additional support providers need to make strong HPV vaccine recommendations.

### Enhancement of healthcare access

This theme captures studies that attempted to reduce existing barriers and magnify accessibility to vaccination. In one study, Schuster focused on families with a six-week-old infant who were visited by a healthcare worker and assisted in identifying possible barriers to vaccination [42]. The immunization rates of African American infants increased after the implementation of case management due to improved access to vaccination for African American parents. The case management approach increased the immunization rate of Black infants in the US by 35% and heightened the number of families that remained up to date with the immunization schedule for infants.

In contrast to this case management intervention, other studies reported low vaccine uptake even after the elimination of barriers to vaccination [42]. One study sought to distribute vaccines across multiple sites that were accessible to Black populations [37]. Plough et al. reported that African Americans experienced low rates of immunization even after the distribution of the H1N1 vaccine at a variety of locations such as parade routes, church parking lots, college campuses, and free vaccine clinics in areas that are conveniently accessed by Black populations [37]. Additionally, an intervention set in a clinical setting within communities of color that provided direct access to the vaccine and removed the cost barriers for vaccine initiation through the Vaccine for Children program found the initiation and completion of vaccination remained below the national average rates [32]. However, while vaccine uptake was poor, Black adolescents attained an improved understanding of the HPV vaccine [32].

### Healthcare provider leadership

Five studies sought to examine the efficacy of vaccination recommendations from an HCP in improving the vaccine acceptance of Black populations [33, 34, 36, 44, 47]. To determine the effectiveness of HCP recommendations, one study administered a survey to Black participants prior to and after an encounter with a HCP to verify that the HPV vaccine was recommended and assessed participants’ impression of how strongly the HCP recommended the HPV vaccine [47]. Results show the reports of participants regarding the HCP who offered the HPV vaccine to participants’ children were positive and associated with higher odds of HPV vaccine uptake.

Black individuals who received a recommendation from an HCP were more likely to obtain a vaccination irrespective of an educational [34] or messaging based intervention [33]. Furthermore, a quantitative study developed a presentation that included statements from an HCP recommending the HPV vaccine after Black female participants in a focus group stated that a strong recommendation from a co-ethnic provider elevated their desire for vaccination [44]. Strengthening of HCP recommendation of vaccines is crucial as many research articles have reported the efficacy of provider recommendation in promoting vaccine acceptance [33, 34, 44, 47]. An online continuing education course providing strategies to make strong HPV vaccine recommendations to East African families in the United States serves as one method to bolster vaccine recommendations [36]. Most of the studies relevant to the theme of HCP leadership identified recommendation from HCPs as a facilitating strategy to increase HPV vaccine uptake.

## Discussion

The results of this review indicate vaccination interventions are largely focused on education, messaging, and HCP leadership, with some, but less, attention aimed at enhancement of health care accessibility, outreach, and multi-dimensional approaches. Our review’s emphasis on education corroborates previous studies that detected a surge in vaccination rates after the implementation of an education-based intervention and HCP vaccine recommendation [49–52]. We add to this work by tracking the types of education interventions used across the studies included. Education-based interventions including videos, PowerPoint presentations, comic books, electronic books, and community forums successfully improved the vaccine literacy of Black participants. For example, 12% of Black study participants received their first dose of the HPV within 7 months after watching a 12-minute presentation about the HPV vaccine [32]. HCPs also received educational strategies that they could utilize to effectively recommend vaccines to patients. According to McFadden et al., online continuing education courses for HCPs offer self-paced learning and increase their ability to effectively recommend HPV vaccines to East African patients as a result of gaining knowledge of East African cultural concerns regarding vaccines [36]. The provision of training for HCPs to adequately address vaccine hesitancy is crucial to ensure vaccine recommendations are effective and culturally appropriate for Black people. Healthcare organizations should consider implementing similar continuing education courses in their respective countries to further enhance vaccine recommendations from HCPs.

While we found an emphasis on education within interventions meant to decrease vaccine hesitancy among Black populations outside of Africa and the Caribbean, this review also suggests such approaches may be limited. Factors contributing to vaccine reluctance among Black populations are not primarily based on a lack of knowledge but instead on concerns regarding vaccine safety, historical injustices, and mistrust of the healthcare system [53].

While educational interventions have been effective in increasing knowledge and vaccine literacy, our findings suggest that knowledge alone may not be sufficient to overcome vaccine hesitancy within Black communities. The varying success rates of interventions highlight the importance of addressing deeper social and psychological factors, such as cultural relevance, trust in healthcare systems, and community engagement. Research shows that culturally tailored interventions, especially those led by trusted community leaders or healthcare providers from similar backgrounds, often achieve greater success because they align with the community’s values and historical experiences with healthcare [5, 12]. Additionally, implementation barriers like limited healthcare access and logistical challenges can reduce the effectiveness of interventions, underlining the need for accessible and adaptable approaches [54]. Integrating behavioral and psychological approaches—such as strategies to build trust, mitigate fears, and leverage social networks—is crucial alongside educational efforts. These factors, especially when rooted in community-specific concerns, can foster psychological safety and encourage vaccine uptake within Black communities [6, 55]. A multifaceted approach is therefore necessary to ensure that interventions extend beyond knowledge dissemination and effectively address vaccine hesitancy among Black populations.

Ensuring equitable access to vaccine services and sites for Black populations is imperative before labeling them as “vaccine hesitant.” Vaccine hesitancy is intricate and requires versatile solutions that can be customized for racial groups. Public health agencies must first comprehend the factors influencing vaccination acceptance among Black individuals as doing so enables interventions to be implemented with greater efficacy.

Factors that influence vaccine hesitancy within Black communities are linked to historical medical research injustices including discrimination and experimentation. For example, the Tuskegee Syphilis Experiment was an unethical study conducted for 40 years on African Americans living with syphilis who were never informed of their diagnosis and instead told by US public health officials that they had ‘bad blood’ [56]. Penicillin existed as a treatment for syphilis but was never offered to the Black participants in the study, who instead received placebos such as mineral supplements. Despite the risk of infecting others and experiencing complications from syphilis such as blindness, deafness, bone deterioration, and death, the participants were never informed of their diagnosis. The Tuskegee study provides the historical context for understanding factors that contribute to Black mistrust of healthcare institutions [53]. Healthcare disparities and structural racism are present day occurrences that are attributed to medical distrust experienced by Black individuals [53].

Multiple factors influence the low vaccine uptake rate within Black communities, including low access to vaccination, perception of vaccine risk, level of trust in HCPs, mistrust of vaccines, disinformation, and discrimination [17, 57]. Efforts need to address factors rooted in social determinants of health and inequalities, including overrepresentation in high-risk jobs, overcrowded living conditions, barriers to healthcare access, experiences of racism within a healthcare institution, and precarious employment, that have a strong impact on the vaccine confidence of people of African and Caribbean descent. Furthermore, anti-Black systemic racism continues to pervade healthcare institutions today. Black populations experience discrimination, misdiagnosed conditions, stereotypes of higher pain tolerance, and denial of their pain by physicians, which further increases their skepticism around any type of vaccine [53, 57].

Therefore, multidimensional interventions may be productive because they can combine an educational approach with the addition of HCP recommendations and outreach activities, which have been shown to be effective in improving vaccination rates [32–34, 36, 39, 44, 45, 47]. A variety of factors contribute to the distrust of vaccines among Black populations, suggesting a combination of versatile interventions targeting different concerns and multiple barriers can be successful in addressing vaccine reluctance. Multimodal interventions could dispel vaccine misinformation, increase accessibility to vaccines, and provide education about vaccines for peoples of African and Caribbean descent. For example, an intervention by Chan et al. revealed the vaccination rates of Black healthcare workers increased after the implementation of multiple approaches such as a hotline, written educational material, department staff hurdles, provision of vaccine stations, and virtual town halls [29]. Multi-dimensional interventions were employed for several purposes in the literature and have been shown to be effective at improving COVID-19 vaccine intentions among older participants [58], promoting mental health wellness [59], enhancing hand hygiene compliance among healthcare workers [60], and maximizing sexual health literacy among Black women [61].

Studies on the use of messages to promote vaccination have yielded mixed results. While Frew et al. found messages propagating the benefits of vaccination (gain frame) and disadvantages of being unvaccinated (loss frame) did not increase immunization rates among Black pregnant participants, Huang et al. revealed a non-narrative message discussing benefits and side effects of the COVID-19 vaccine was effective at positively affecting vaccine intentions [33, 43]. In contrast to the findings of Frew et al., the employment of message framing in Kasting et al. indicated participants who received either a gain or loss frame message about vaccination received more doses than those in the control group [33, 49]. Future studies should consider acknowledging unjust medical research within vaccine interventions as medical mistrust is a facet of vaccine hesitancy among Black people. Messages acknowledging historical injustices were more effective at encouraging Black populations to get vaccinated than messages emphasizing safety and general information about COVID-19 vaccines [31].

Messaging-based and education-focused interventions have not revealed the increased risk of comorbidities that African Americans face when they contract infections such as COVID-19 while living with a pre-existing chronic condition. Promoting the negative outcomes resulting from combining a chronic illness with a viral infection could increase vaccine acceptance among Black people [31]. Future messaging-based interventions could include information about the methodology used to develop and test vaccine efficacy, possible side effects, a question-and-answer format, and a frequently asked questions section.

Teteh et al. suggest community forums can facilitate dialogue with community members and serve as an education strategy to enhance the understanding of vaccine functionality [40]. Their study expands the understanding of conducting health forums to provide community education about vaccines. The inclusion of a pharmaceutical expert who described the process of vaccine development and methods to verify vaccine safety reassured Black community members about the effectiveness of vaccines but also fostered trust. This model has the potential to establish a connection between education, messaging, and community engagement, thus enabling efficacious interventions.

Community engagement in research can enhance the ability of a community to address its own health needs and health disparities while ensuring that researchers understand the priorities of the community [62]. This review identified only one community-based intervention; however, the literature propagates the significant benefit it holds for minority communities. Community-based interventions are a critical component and Black community stakeholders have already begun taking action within their communities to elevate vaccine confidence. Researchers should seek to collaborate with Black organizations and community leaders to develop well-informed interventions and share existing evidence.

The implementation of community forums is not limited to vaccination enhancement. For instance, community forums have been utilized to increase awareness about stroke and stroke prevention [63], to heighten community member knowledge of the opioid crisis [64] and to provide social support to parents of children diagnosed with Type 1 diabetes [65]. The findings of this review are consistent with the results of Bharmal et al. that indicate community forums are an effective method for enhancing knowledge and awareness post-intervention [63]. Community-engagement approaches are better able to overcome challenges that influence vaccine hesitancy when rooted in principles of power sharing, capacity building, authentic partnership, collective learning, and co-creation [66]. Therefore, health research teams and public health practitioners can attempt to rebuild trust with Black communities by collaborating on the creation of health interventions to ensure they are culturally appropriate and informed. This may offer avenues to increase Black people’s access to health care more generally in countries outside of Africa and the Caribbean.

Finally, we found HCP leadership is associated with improved coverage for many vaccines, including influenza, HPV, COVID-19, and pneumococcal diseases among people of African and Caribbean descent [9, 33, 34, 36, 44]. An imperative is that future interventions include vaccine recommendations from HCP in alternative methods distinct from clinical visits as Black populations lacking health insurance cannot easily access HCPs. For example, interventions consisting of social media posts, community forums, presentations, media campaigns, and vaccine distribution sites could include vaccine recommendation messages, videos, and images of an HCP [35, 37, 39].

This study adds to prior evidence regarding the positive influence of provider recommendations in driving acceptance of vaccinations. A United States study by Nguyen et al. reported that adults (77.6%) who received a HCP recommendation were highly likely to receive more than one dose of COVID-19 vaccine than those who never received a recommendation (61.9%) [67]. The existing literature has also provided strategies for effective recommendations from HCPs such as maintenance of a trustworthy relationship with patients and tailoring recommendations to the concerns of the patient [36, 68, 69]. Recommendations from HCPs are a major driver of vaccine acceptance among vaccine-hesitant patients. This review suggests HCPs take a proactive approach in addressing vaccine hesitancy. One strategy is to visit community centers to reach a large number of Black individuals as primary clinic sites are not a feasible option for enhancing vaccine rates due to limited access to clinics and challenges with transportation. However, future studies should examine the extent of vaccine hesitancy among HCPs themselves. Several studies discovered that HCPs who were already vaccinated themselves or had intentions for vaccination were more likely to recommend vaccination to their patients [70–73]. To ensure recommendations will be efficacious, vaccine recommendations should occur after providers have demonstrated an acceptance of vaccines.

Additionally, HCPs can utilize the LEAPS (listening, empathy, articulation, persuasion, and stewardship) communication framework [74] (which has already been implemented in Canada) during their interactions with Black patients to recommend a vaccine [16]. The LEAPS communication framework recommends that providers listen and learn about the patients’ lived experiences; engage and empower patients by respecting their self-determination; ask and acknowledge patients fears and concerns; paraphrase and provide vaccination information; and spark and support future patient engagement and community partnerships as necessary [16]. This review supports the implementation of the LEAPS communication framework within outreach interventions and utilization by HCPs when recommending vaccines to Black patients.

This study revealed a limited distribution of research studies on interventions employed to address vaccine hesitancy among Black populations geographically, as the majority of current studies originate in the United States. This indicates a gap concerning interventions aimed at enhancing vaccine confidence in other countries including Canada, France, Australia, and the United Kingdom. This review provides information on existing interventions that Western nations and European countries with Black populations can draw lessons from to heighten vaccine confidence. The findings of this review also reveal a lack of health policies specifically targeting Black populations. Finally, a dearth of qualitative research that examines practices that can promote vaccine confidence among Black people exists; most of the studies reviewed were quantitative (*n* = 17). Thus, while research shows an elevated prevalence rate of infectious diseases among Black populations, it is still unclear which contextual factors influence the hesitation around vaccines.

The knowledge gained from this review regarding various intervention approaches will have significant implications for governments, public health organizations, and HCPs in their response to the complex issue of vaccine hesitancy within Black communities. One research study provided a catalog of interventions utilized to address vaccine hesitancy in Europe among general populations [75]. However, there is no review apart from the present study examining vaccine confidence-building interventions developed specifically for Black populations. Previous public health interventions have been designed for entire populations, but tailored interventions are needed for Black populations as they are disproportionately burdened by infectious diseases. The interventions outlined in this review can be adapted to different contexts and various types of vaccines. Government health departments and public health agencies are urged to work towards increasing vaccine confidence within Black communities. A unique contribution of this study lies in its comprehensive delineation of effective vaccine interventions that promote vaccine confidence among Black populations.

## Conclusion

Black populations are disproportionately burdened by high numbers of COVID-19, influenza, and cervical cancer cases but low vaccination uptake. The reduction of infection disparities among African Americans requires increasing vaccination confidence. This review sought to map current research on measures that address hesitancy related to vaccines in Black populations living outside of African and Caribbean countries and identify gaps in research on vaccine-related interventions that would decrease the distrust of vaccines. Qualitative data concerning interventions that address Black people’s experiences with reluctance related to vaccines and the building of trust within interventions are urgently needed. This review identified elevated vaccination uptake among Black participants after the implementation of education-based interventions, multidimensional approaches, and HCP vaccine recommendations. Intervention studies are needed in Canada, the United Kingdom, France, and Australia to bolster the vaccine confidence of Black populations in each country as the majority of studies included in this review were conducted in the United States. This review can serve to support the implementation of evidence-based interventions that will reduce mistrust of vaccines and tackle concerns causing hesitancy around vaccines.

### Limitations of the study

This study has several limitations that should be acknowledged. First, while we aimed for a comprehensive review, the inclusion criteria limited the scope to articles published in English, which may exclude relevant studies published in other languages. Another limitation is the lack of a formal quality appraisal of the included studies, a common constraint in scoping reviews. This may affect the robustness of the findings, as studies of varying methodological quality were included without differentiation. The studies reviewed here also varied widely in their design, participant demographics, and contexts, which makes direct comparisons challenging.

Lastly, most of the included studies were conducted in the United States, with limited representation from other regions, such as Canada, the UK, and Australia, which may impact the generalizability of findings across different Black communities worldwide. Future research should address these gaps to provide a more globally representative understanding of interventions that can effectively address vaccine hesitancy.

### Policy recommendations

To effectively address vaccine hesitancy among Black populations, public health authorities should create and implement vaccination campaigns specifically tailored to these communities. Successful campaigns in other settings demonstrate the impact of culturally relevant messaging that acknowledges both historical and contemporary injustices in healthcare, which contribute to mistrust [76, 77]. Incorporating elements such as the safety and efficacy of vaccines, alongside addressing specific community concerns, has proven effective in similar contexts, and can enhance vaccine confidence among Black populations [6, 54].

Governments and funding agencies should prioritize community-based interventions, such as forums, mobile clinics, and partnerships with trusted local leaders and organizations. Examples from other communities show that these grassroots approaches can successfully build trust, engage communities, and provide accessible vaccination services [76, 78]. Additionally, training programs on cultural competency and effective communication should be mandated for healthcare providers (HCPs), equipping them to address unique concerns and barriers faced by Black individuals. Improved understanding and tailored communication from HCPs can have a significant impact on vaccine uptake [55].

Additionally, policy efforts should prioritize interventions that consider socioeconomic disparities and migration-related challenges. Many Black individuals, particularly recent migrants and those from lower-income backgrounds, face structural barriers such as limited access to healthcare facilities, lack of transportation, and financial constraints. Policy recommendations should therefore include expanding vaccination access beyond traditional healthcare settings, such as through mobile clinics, community centers, and faith-based organizations, which are more accessible to those with limited resources. Additionally, providing vaccination services at low or no cost and implementing policies that ensure language accessibility and culturally sensitive practices can bridge gaps in vaccine access. Training healthcare providers in culturally responsive communication, particularly for diverse migrant groups, is also essential to build trust and address historical mistrust in healthcare systems. These policy initiatives can reduce structural inequities in healthcare access, making vaccination efforts more inclusive and effective in reaching Black communities with tailored, equitable interventions.

Policymakers should also address structural barriers to vaccination, including healthcare access, transportation, and insurance coverage. Studies show that providing vaccines at convenient locations and at no cost increases vaccination rates among marginalized populations [77]. Public health practitioners should utilize educational materials that resonate with Black communities, including testimonials from trusted figures, information about vaccine safety, and the benefits of vaccination in preventing severe health outcomes.

A multi-component approach combining education, outreach, and HCP recommendations has been effective in increasing vaccine uptake in other communities and can be adapted to meet the unique needs of Black populations. Leveraging media and technology, such as social media campaigns, online educational videos, and virtual town halls, can expand the reach and impact of these messages, as demonstrated in various successful public health campaigns [6, 54]. These platforms allow for rapid dissemination of accurate information and foster engagement with a broader audience within the Black community.

By implementing these evidence-based policy and practice recommendations, public health authorities and HCPs can better address vaccine hesitancy, ultimately reducing health disparities and improving overall community health among Black populations.

### Future research directions

Future research should prioritize a deeper understanding of the complex factors contributing to vaccine hesitancy among Black populations, with a particular focus on qualitative studies that investigate contextual factors, such as lived experiences, perceptions, and beliefs. These insights will be instrumental in developing tailored and effective interventions that resonate with Black communities. Immediate research needs include exploring the impact of healthcare provider (HCP) recommendations delivered through non-traditional channels outside clinical visits. Given the barriers many Black individuals face in accessing healthcare, future studies should examine the effectiveness of alternative settings—such as community centers, religious institutions, and social media platforms—in influencing vaccine acceptance and uptake.

A critical area for investigation is the effectiveness of community-based participatory research (CBPR) approaches in vaccine interventions. CBPR emphasizes active community involvement, ensuring interventions are culturally aligned and community-informed. Research should evaluate CBPR’s impact on vaccine confidence and uptake among Black populations, as well as its potential for fostering trust between healthcare systems and communities.

Longitudinal studies are also essential to assess the sustained impact of multi-component interventions on vaccine uptake and health outcomes. Tracking participants over time can provide insights into the durability of intervention effects and reveal factors supporting long-term vaccine behavior changes. Comparative studies across regions and healthcare systems are recommended to understand how local contexts influence the effectiveness of interventions.

Finally, there is a need to investigate the effects of policy changes on vaccine hesitancy and uptake within Black populations, especially policies aimed at mitigating structural barriers, such as improving healthcare access, transportation, and insurance coverage. Examining how these policy interventions interact with community-based efforts will be invaluable in identifying comprehensive, multifaceted strategies for addressing vaccine hesitancy and enhancing public health outcomes. Addressing these research priorities will contribute to a more nuanced understanding of vaccine hesitancy and inform the development of culturally relevant, effective interventions for Black populations, ultimately advancing health equity and reducing disparities.

## Electronic supplementary material

Below is the link to the electronic supplementary material.

Supplementary Material 1

## Data Availability

No datasets were generated or analysed during the current study.

## References

[1] World Health Organization. Understanding the behavioural and social drivers of vaccine uptake: WHO position paper – may 2022. Wkly Epidemiol Rec. 2022;97:209–24.

[2] MacDonald NE, SAGE Working Group on Vaccine Hesitancy. Vaccine hesitancy: definition, scope and determinants. Vaccine. 2015;33:4161–4. 25896383 10.1016/j.vaccine.2015.04.036

[3] World Health Organization. Ten threats to global health in 2019. https://www.who.int/news-room/spotlight/ten-threats-to-global-health-in-2019 Accessed 23 Jul 2024.

[4] Laurencin CT. Addressing justified vaccine hesitancy in the black community. J Racial Ethn Health Disparities. 2021;8:543–6. 33783755 10.1007/s40615-021-01025-4 PMC8009077

[5] Bogart LM, Dong LU, Gandhi P, Ryan S, Smith TL, Klein DJ et al. What contributes to COVID-19 vaccine hesitancy in Black communities, and how can it be addressed? Santa Monica, CA; 2021.

[6] Dubé E, Laberge C, Guay M, Bramadat P, Roy R, Bettinger JA. Vaccine hesitancy. Hum Vaccin Immunother. 2013;9:1763–73. 23584253 10.4161/hv.24657 PMC3906279

[7] Ledur J. COVID-19 is affecting Black, Indigenous, Latinx, and other people of color the most. COVID Racial Data Tracker. 2020;:1–7. https://covidtracking.com/race

[8] Egede LE, Walker RJ. Structural racism, social risk factors, and Covid-19 — a dangerous convergence for Black americans. N Engl J Med. 2020;11:1089–92. 10.1056/NEJMp2023616 PMC7747672 32706952

[9] Fu J, Reid SA, French B, Hennessy C, Hwang C, Gatson NT, et al. Racial disparities in COVID-19 outcomes among black and white patients with cancer. JAMA Netw Open. 2022;5:1–14. 10.1001/jamanetworkopen.2022.4304 PMC8961318 35344045

[10] Office for National Statistics. Why have Black and South Asian people been hit hardest by COVID-19? Office for National Statistics. 2020. https://www.ons.gov.uk/peoplepopulationandcommunity/healthandsocialcare/conditionsanddiseases/articles/whyhaveblackandsouthasianpeoplebeenhithardestbycovid19/2020-12-14. Accessed 24 Jun 2024.

[11] Statistics Canada. COVID-19 vaccine willingness among Canadian population groups. Statistics Canada. 2021. https://www150.statcan.gc.ca/n1/pub/45-28-0001/2021001/article/00011-eng.htm. Accessed 24 Jun 2024.

[12] Quinn SC, Lama Y, Jamison A, Freimuth V, Shah V. Willingness of Black and White adults to accept vaccines in development: an exploratory study using national survey data. Am J Health Promotion. 2021;35:571–9. 10.1177/0890117120979918 33356411

[13] Ali AK, Celentano PL. Addressing vaccine hesitancy in the post-truth era. Eurohealth (Lond). 2017;23:16–20.

[14] Castillo JC, Ahuja A, Athey S, Baker A, Budish E, Chipty T et al. Market design to accelerate COVID-19 vaccine supply. Science (1979). 2021;371. 10.1126/science.abg0889 33632897

[15] Force BST, Black Scientists’ Task Force on Vaccine Equity. 2021. https://www.toronto.ca/news/black-scientists-task-force-on-vaccine-equity/#:~:text=The Task Force will present,City by April 30%2 C 2021.&text = Akwatu Khenti-,Dr.,Task Force on Vaccine Equity. Accessed 28 May 2024.

[16] Eissa A, Lofters A, Akor N, Prescod C, Nnorom O. Increasing SARS-CoV-2 vaccination rates among black people in Canada. CMAJ. 2021;193:E1220–1. 34373272 10.1503/cmaj.210949 PMC8367428

[17] Dada D, Djiometio JN, McFadden SAM, Demeke J, Vlahov D, Wilton L, et al. Strategies that promote equity in COVID-19 vaccine uptake for black communities: a review. J Urb Health. 2022;99:15–27. 10.1007/s11524-021-00594-3 PMC8751469 35018612

[18] Adeagbo M, Olukotun M, Musa S, Alaazi D, Allen U, Renzaho AMN et al. Improving COVID-19 vaccine uptake among black populations: a systematic review of strategies. Int J Environ Res Public Health. 2022;19. 10.3390/ijerph191911971 PMC9565689 36231270

[19] Opel DJ, Mangione-Smith R, Robinson JD, Heritage J, DeVere V, Salas HS, et al. The influence of provider communication behaviors on parental vaccine acceptance and visit experience. Am J Public Health. 2015;105:1998–2004. 25790386 10.2105/AJPH.2014.302425 PMC4566548

[20] Escoffery C, Petagna C, Agnone C, Perez S, Saber LB, Ryan G, et al. A systematic review of interventions to promote HPV vaccination globally. BioMed Central; 2023. 10.1186/s12889-023-15876-5 PMC10308645 37386430

[21] Brewer NT, Chapman GB, Rothman AJ, Leask J, Kempe A. Increasing vaccination: putting psychological science into action. Psychol Sci Public Interest. 2017;18:149–207. 29611455 10.1177/1529100618760521

[22] Betsch C, Brewer NT, Brocard P, Davies P, Gaissmaier W, Haase N, et al. Opportunities and challenges of web 2.0 for vaccination decisions. Vaccine. 2012;30:3727–33. 22365840 10.1016/j.vaccine.2012.02.025

[23] Larson HJ, de Figueiredo A, Xiahong Z, Schulz WS, Verger P, Johnston IG, et al. The state of vaccine confidence 2016: global insights through a 67-country survey. EBioMedicine. 2016;12:295–301. 27658738 10.1016/j.ebiom.2016.08.042 PMC5078590

[24] Arksey H, O’Malley L. Scoping studies: towards a methodological framework. Int J Social Res Methodology: Theory Pract. 2005;8:19–32.

[25] Covidence. A step-by-step guide to completing your systematic review. 2024. https://www.covidence.org/wp-content/uploads/2024/01/A-step-by-step-guide.pdf

[26] Page MJ, McKenzie JE, Bossuyt PM, Boutron I, Hoffmann TC, Mulrow CD et al. The PRISMA 2020 statement: an updated guideline for reporting systematic reviews. BMJ. 2021;372. 10.1136/bmj.n71 PMC8005924 33782057

[27] Braun V, Clarke V. Using thematic analysis in psychology. Qual Res Psychol. 2006;3:77–101.

[28] Alio AP, Lewis CA, Bunce CA, Wakefield S, Thomas WG, Sanders E, et al. Capacity building among African American faith leaders to promote HIV prevention and vaccine research. Prog Community Health Partnersh. 2014;8:305–16. 25435557 10.1353/cpr.2014.0050

[29] Chan WY, Entwisle C, Ercoli G, Ramos-Sevillano E, McIlgorm A, Cecchini P et al. Correction for Chan A Novel, Multiple-Antigen Pneumococcal Vaccine Protects against Lethal Streptococcus pneumoniae Challenge. Infect Immun. 2022;90:e0063921. 10.1128/IAI.00639-21 PMC8876107 35076290

[30] Chapman E, Venkat P, Ko E, Orezzoli JP, Del Carmen M, Garner EIO. Use of multimedia as an educational tool to improve human papillomavirus vaccine acceptability-A pilot study. Gynecol Oncol. 2010;118:103–7. 20457469 10.1016/j.ygyno.2010.04.010

[31] Dhanani LY, Franz B. An experimental study of the effects of messaging strategies on vaccine acceptance and hesitancy among Black americans. Prev Med Rep. 2022;27:101792. 35433238 10.1016/j.pmedr.2022.101792 PMC9006422

[32] Diclemente RJ, Murray CC, Graham T, Still J. Overcoming barriers to HPV vaccination: a randomized clinical trial of a culturally-tailored, media intervention among African American girls. Hum Vaccin Immunother. 2015;11:2883–94. 26378650 10.1080/21645515.2015.1070996 PMC5054780

[33] Frew PM, Saint-Victor DS, Owens LE, Omer SB. Socioecological and message framing factors influencing maternal influenza immunization among minority women. Vaccine. 2014;32:1736–44. 24486366 10.1016/j.vaccine.2014.01.030

[34] Kriss JL, Frew PM, Cortes M, Malik FA, Chamberlain AT, Seib K, et al. Evaluation of two vaccine education interventions to improve pertussis vaccination among pregnant African American women: a randomized controlled trial. Vaccine. 2017;35:1551–8. 28216190 10.1016/j.vaccine.2017.01.037 PMC5570451

[35] Lashuay N, Tjoa T, Zuniga De Nuncio ML, Franklin MR, Elder J, Jones M. Exposure to immunization media messages among African American parents. Prev Med (Baltim). 2000;31:522–8. 10.1006/pmed.2000.0745 11071832

[36] McFadden SAM, Ko LK, Shankar M, Ibrahim A, Berliner D, Lin J, et al. Development and evaluation of an online continuing education course to increase healthcare provider self-efficacy to make strong HPV vaccine recommendations to east African immigrant families. Tumour Virus Res. 2021;11:200214. 33647533 10.1016/j.tvr.2021.200214 PMC7944093

[37] Plough A, Bristow B, Fielding J, Caldwell S, Khan S. Pandemics and health equity: lessons learned from the H1N1 response in Los Angeles County. J Public Health Manage Pract. 2011;17:20–7. 10.1097/PHH.0b013e3181ff2ad7 21135657

[38] Shin MB, Ko LK, Ibrahim A, Mohamed FB, Lin J, Celentano I, et al. The impact of a comic book intervention on east African-American adolescents’ HPV vaccine-related knowledge, beliefs and intentions. J Immigr Minor Health. 2022;24:1489–500. 35357620 10.1007/s10903-022-01359-z PMC10129048

[39] Staples JN, Wong MS, Rimel BJ. An educational intervention to improve human papilloma virus (HPV) and cervical cancer knowledge among African American college students. Gynecol Oncol. 2018;149:101–5. 29605043 10.1016/j.ygyno.2017.10.015

[40] Teteh DK, Dawkins-Moultin L, Robinson C, LaGroon V, Hooker S, Alexander K, et al. Use of community forums to increase knowledge of HPV and cervical cancer in African American communities. J Community Health. 2019;44:492–9. 30989454 10.1007/s10900-019-00665-2

[41] Tiro JA, Sanders JM, Pruitt SL, Stevens CF, Skinner CS, Bishop WP, et al. Promoting HPV vaccination in safety-net clinics: a randomized trial. Pediatrics. 2015;136:850–9. 26482674 10.1542/peds.2015-1563 PMC7313721

[42] Schuster M, Wood D, Duan N, Grabowsky M, Donald-Sherbourne C, Mazel RM, et al. Increasing immunization rates among inner-city, African American children. JAMA. 1998;279:29–34. 9424040 10.1001/jama.279.1.29

[43] Huang Y, Green MC. Reducing COVID-19 vaccine hesitancy among African americans: the effects of narratives, character’s self-persuasion, and trust in science. J Behav Med. 2023;46:290–302. 35305206 10.1007/s10865-022-00303-8 PMC8933767

[44] Chu H, Ko LK, Ibrahim A, Bille Mohamed F, Lin J, Shankar M, et al. The impact of an educational forum intervention on east African mothers’ HPV vaccine-related knowledge, attitudes, and intentions to vaccinate their adolescent children. Vaccine. 2021;39:3767–76. 34053792 10.1016/j.vaccine.2021.05.029 PMC9984200

[45] Gadarian SK, Goodman SW, Michener J, Nyhan B, Pepinsky TB. Information from same-race/ethnicity experts online does not increase vaccine interest or intention to vaccinate. Milbank Q. 2022;100:492–503. 35315950 10.1111/1468-0009.12561 PMC9111148

[46] Alsan M, Garrick O, Graziani G. Does diversity matter for health? Experimental evidence from Oakland. Am Econ Rev. 2019;109:4071–111.

[47] Fu LY, Zimet GD, Latkin CA, Joseph JG. Associations of trust and healthcare provider advice with HPV vaccine acceptance among African American parents. Vaccine. 2017;35:802–7. 28063706 10.1016/j.vaccine.2016.12.045 PMC5290730

[48] Alsan M, Chandra A, Simon K. The great unequalizer: initial Health effects of COVID-19 in the United States. J Economic Perspect. 2021;35:25–46.

[49] Kasting ML, Head KJ, Cox D, Cox AD, Zimet GD. The effects of message framing and healthcare provider recommendation on adult hepatitis B vaccination: a randomized controlled trial. Prev Med (Baltim). 2019;127 May:105798. 10.1016/j.ypmed.2019.105798 PMC6744972 31404569

[50] Dempsey AF, Pyrznawoski J, Lockhart S, Barnard J, Campagna EJ, Garrett K, et al. Effect of a health care professional communication training intervention on adolescent human papillomavirus vaccination a cluster randomized clinical trial. JAMA Pediatr. 2018;172:1–9. 10.1001/jamapediatrics.2018.0016 PMC5875329 29507952

[51] Smulian EA, Mitchell KR, Stokley S. Interventions to increase HPV vaccination coverage: a systematic review. Hum Vaccin Immunother. 2016;12:1566–88. 26838959 10.1080/21645515.2015.1125055 PMC4964671

[52] Lott BE, Okusanya BO, Anderson EJ, Kram NA, Rodriguez M, Thomson CA et al. Interventions to increase uptake of human papillomavirus (HPV) vaccination in minority populations: a systematic review. Prev Med Rep. 2020;19 June 2020:101163. 10.1016/j.pmedr.2020.101163 PMC7372149 32714778

[53] Bajaj SS, Stanford FC. Beyond Tuskegee — vaccine distrust and everyday racism. N Engl J Med. 2021;384:4–5. 10.1056/NEJMpv2035827 PMC9908408 33471971

[54] Viswanath K, Ackerson LK. Race, ethnicity, language, social class, and health communication inequalities: a nationally- representative cross-sectional study. PLoS ONE. 2011;6. 10.1371/journal.pone.0014550 PMC3022647 21267450

[55] Betsch C, Böhm R, Chapman GB. Using behavioral insights to increase vaccination policy effectiveness. Policy Insights Behav Brain Sci. 2015;2:61–73.

[56] Alsan M, Wanamaker M. Tuskegee and the health of black men. Q J Econ. 2018;133:407–55. 30505005 10.1093/qje/qjx029 PMC6258045

[57] Bleser WK, Miranda PY, Jean-Jacques M. Racial/ethnic disparities in influenza vaccination of chronically-ill US adults: The mediating role of perceived discrimination in healthcare (Medical Care (2016) 54 (570–577)). Med Care. 2016;54:570–577. 10.1097/MLR.0000000000000544 PMC6060271 27172536

[58] Johnson V, Butterfuss R, Kim J, Orcutt E, Harsch R, Kendeou P. The ‘Fauci Effect’: reducing COVID-19 misconceptions and vaccine hesitancy using an authentic multimodal intervention. Contemp Educ Psychol. 2022;70:102084. 35765462 10.1016/j.cedpsych.2022.102084 PMC9221368

[59] Przybylko G, Morton D, Kent L, Morton J, Hinze J, Beamish P, et al. The effectiveness of an online interdisciplinary intervention for mental health promotion: a randomized controlled trial. BMC Psychol. 2021;9:1–19. 33975645 10.1186/s40359-021-00577-8 PMC8111974

[60] Watson JA. Role of a multimodal educational strategy on health care workers’ handwashing. Am J Infect Control. 2016;44:400–4. 26739638 10.1016/j.ajic.2015.10.030

[61] Bondi E, Craddock J, Funke R, Legendre C, Tiwari V. Maximizing the spread of sexual health information in a multimodal communication network of young black women. Artif Intell Social Work. 2018;:93–118.

[62] Ahmed SM, Palermo AGS. Community engagement in research: frameworks for education and peer review. Am J Public Health. 2010;100:1380–7. 20558798 10.2105/AJPH.2009.178137 PMC2901283

[63] Bharmal N, Lucas-Wright AA, Vassar SD, Jones F, Jones L, Wells R, et al. A community engagement symposium to prevent and improve stroke outcomes in diverse communities. Prog Community Health Partnersh. 2016;10:149–58. 27018364 10.1353/cpr.2016.0010 PMC4943874

[64] Palombi L, Olivarez M, Bennett L, Hawthorne AN. Community forums to address the opioid crisis: an effective grassroots approach to rural community engagement. Subst Abuse. 2019;13:1–7. 10.1177/1178221819827595 PMC6378421 30799927

[65] Balkhi AM, Reid AM, McNamara JP, Geffken GR. The diabetes online community: the importance of forum use in parents of children with type 1 diabetes. Pediatr Diabetes. 2014;15:408–15. 24372986 10.1111/pedi.12110

[66] Wallerstein NB, Duran B. Using community-based participatory research to address health disparities. Health Promot Pract. 2006;7:312–23. 16760238 10.1177/1524839906289376

[67] Nguyen KH, Yankey D, Lu P, Kriss JL, Brewer NT, Razzaghi H et al. Report of health care provider recommendation for COVID-19 vaccination among adults, by recipient COVID-19 vaccination status and attitudes — United States, April–September. 2021. 2021. 10.15585/mmwr.mm7050a1 PMC8675662 34914669

[68] Healy CM, Pickering LK. How to communicate with vaccine-hesitant parents. Pediatrics. 2011;127 SUPPL. 1:127–33. 10.1542/peds.2010-1722S 21502238

[69] Leask J, Kinnersley P, Jackson C, Cheater F, Bedford H, Rowles G. Communicating with parents about vaccination: a framework for health professionals. BMC Pediatr. 2012;12. 10.1186/1471-2431-12-154 PMC3480952 22998654

[70] Veenstra G, Patterson AC. Black–white health inequalities in Canada. J Immigr Minor Health. 2016;18:51–7. 25894533 10.1007/s10903-014-0140-6

[71] Zhang J, While AE, Norman IJ. Nurses’ knowledge and risk perception towards seasonal influenza and vaccination and their vaccination behaviours: a cross-sectional survey. Int J Nurs Stud. 2011;48:1281–9. 21474136 10.1016/j.ijnurstu.2011.03.002

[72] Livni G, Chodik G, Yaari A, Tirosh N, Ashkenazi S. Attitudes, knowledge and factors related to acceptance of influenza vaccine by pediatric healthcare workers. J Pediatr Infect Dis. 2008;3:111–7.

[73] Askarian M, Khazaeipour Z, McLaws ML. Facilitators for influenza vaccination uptake in nurses at the Shiraz University of Medical Sciences. Public Health. 2011;125:512–7. 21798568 10.1016/j.puhe.2011.03.012

[74] Lukaszewski JE. Lukaszewski on crisis communication: what your CEO needs to know about reputation risk and crisis management. Brookfield, CT: Rothstein Publishing; 2015.

[75] European Centre for Disease Prevention and Control (ECDC). Catalogue of interventions addressing vaccine hesitancy. Stockholm; 2017.

[76] Bogart LM, Ojikutu BO, Tyagi K, Klein DJ, Mutchler MG, Dong L et al. COVID-19 related medical mistrust, health impacts, and potential vaccine hesitancy among Black Americans living with HIV. J Acquir Immune Defic Syndr (1988). 2021;86:200–7. 10.1097/QAI.0000000000002570 PMC7808278 33196555

[77] Quinn SC, Jamison AM, Freimuth VS, An J, Hancock GR, Musa D. Exploring racial influences on flu vaccine attitudes and behavior: results of a national survey of White and African American adults. Vaccine. 2017;35:1167–74. 28126202 10.1016/j.vaccine.2016.12.046 PMC5839483

[78] Freimuth VS, Quinn SC, Thomas SB, Cole G, Zook E, Duncan T. African americans’ views on research and the Tuskegee Syphilis study. Soc Sci Med. 2001;52:797–808. 11218181 10.1016/s0277-9536(00)00178-7

